# Cardiac CT in the era of artificial intelligence: precision imaging, treatment guidance and optimised risk stratification for coronary artery disease

**DOI:** 10.1136/openhrt-2025-003505

**Published:** 2025-11-13

**Authors:** Zhiqi Zhong, Xu Dai, Lihua Yu, Yarong Yu, Jiajun Yuan, Yidan Xu, Jiayin Zhang

**Affiliations:** 1 Radiology, Shanghai General Hospital, Shanghai, China; 2 Faculty of Medical Imaging Technology, College of Health Science and Technology, Shanghai Jiao Tong University School of Medicine, Shanghai, China

**Keywords:** Coronary Artery Disease, Multidetector Computed Tomography, Diagnostic Imaging

## Abstract

Coronary artery disease (CAD) remains a leading cause of morbidity and mortality worldwide, and CT imaging plays a crucial role in its diagnosis and management. However, the clinical use of CT is limited by factors, such as suboptimal image quality, diagnostic complexity and the labour-intensive nature of parameter evaluation. Artificial intelligence (AI) is increasingly transforming many areas of medicine. Its integration into CAD CT imaging can enhance image postprocessing, streamline anatomical and functional analyses, support treatment planning and improve risk prediction. This review summarises recent advances in these AI applications, aiming to promote their practical adoption and further development.

## Introduction

 Despite advances in preventive strategies, coronary artery disease (CAD) remains a leading cause of global morbidity and mortality.^1^ Cardiac CT and coronary CT angiography (CCTA) serve as reliable first-line imaging modalities for evaluating CAD severity. Their primary functions include quantifying coronary artery calcium scores (CACS), assessing coronary stenosis and conducting quantitative analyses of atherosclerotic plaques.^2 3^ Additionally, emerging techniques and parameters, such as CT-derived fractional flow reserve (CT-FFR), which estimates lesion-specific ischemia through computational modelling or machine learning (ML), myocardial perfusion assessed via CT myocardial perfusion imaging (CT-MPI) and myocardial fibrosis identified through late iodine enhancement (LIE), visualising delayed iodine washout, are improving the diagnostic and prognostic value of CT in CAD.^4^,^6^

**Figure 1:**
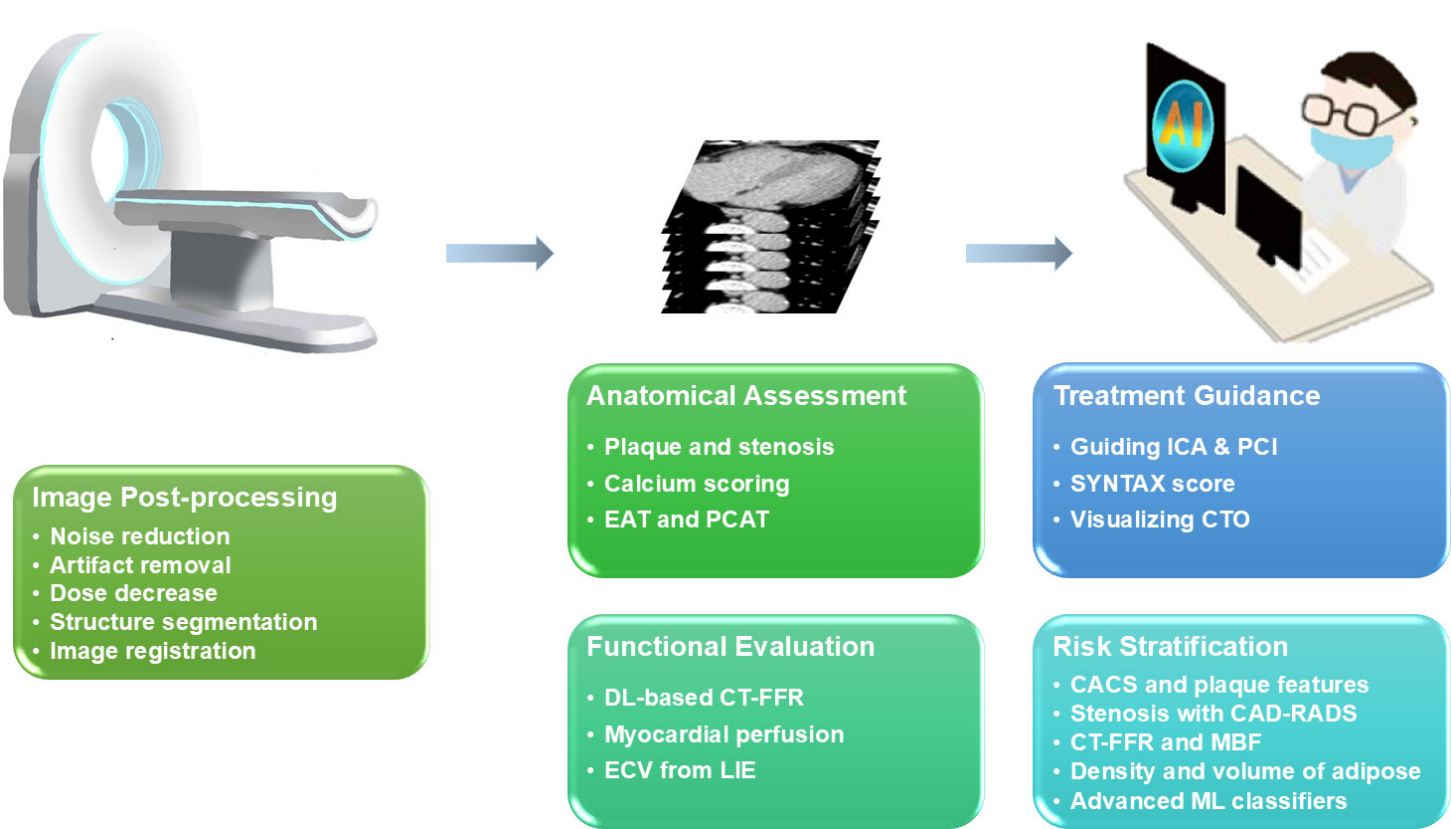
Overview of AI-enhanced cardiac CT in coronary artery disease. AI, artificial intelligence; CACS, coronary artery calcium scores; CAD-RADS, coronary artery disease reporting and data system; CT-FFR, CT-derived fractional flow reserve; CTO, chronic total occlusion; EAT, epicardial adipose tissue; DL, deep learning; ECV, extracellular volume; ICA, invasive coronary angiography; LIE, late iodine enhancement; MBF, myocardial blood flow; ML, machine learning; PCAT, pericoronary adipose tissue; PCI, percutaneous coronary intervention; SYNTAX, synergy between percutaneous coronary intervention with taxus and cardiac surgery.

Cardiac CT use is constrained by suboptimal image quality under conventional reconstruction, diagnostic complexity and the labour-intensive nature of manual parameter extraction. These limitations highlight the need for artificial intelligence (AI) to enhance the accuracy and efficiency of cardiac CT.^7^,^9^ Moreover, AI has the potential to strengthen the clinical utility of CT imaging parameters for CAD by advancing diagnostic precision, treatment decision-making and risk stratification.^10^ Given the rapid progress in AI, this review focuses on recent major AI applications in CAD-related CT imaging. It examines developments across five key domains: image postprocessing, anatomical assessment, functional evaluation, treatment guidance and risk stratification (figure 1). The overall aim was to provide current insights and promote continued innovation and clinical adoption of AI.

### AI and image postprocessing

In recent years, numerous deep learning (DL)-based image postprocessing techniques have been developed (table 1). Among these, DL-based noise reduction has been applied across multiple anatomical regions,^11^ with major progress achieved in cardiac CT. Matsuyama *et al*^12^ validated the noise-reduction performance of the DL image reconstruction (DLIR) tool Advanced Intelligent Clear-IQ Engine (AiCE) and the high-resolution DLIR tool Precise IQ Engine (PIQE) for CCTA, both in vitro and in vivo. Regardless of area-detector CT or ultra-high-resolution CT use, PIQE achieved significantly lower noise levels compared with AiCE and various iterative reconstruction (IR) methods.^12^ However, AiCE and PIQE are vendor-specific, whereas ClariCT.AI is vendor-neutral, making its application more broadly generalisable.^11^ Hong *et al*^13^ developed a CCTA denoising technique that combines ClariCT.AI with IR, achieving significant noise reduction and improved contrast-to-noise and signal-to-noise ratios. Although image sharpness decreased after denoising, diagnostic accuracy remained stable.^13^ Denoising techniques have also been extended to additional devices and sequences. Photon-counting CCTA (PC-CCTA) offers high spatial resolution but is prone to excessive noise.^14^ To address this, Chang *et al*^14^ developed the prior-information-enabled neural network, named Pie-Net, which uses low-noise virtual monoenergetic images (VMIs) as priors to denoise high-resolution VMIs. This model achieved high image fidelity with a 95%±1% noise reduction compared with filtered back projection and a 60%±8% reduction versus IR. Incorporating low-noise VMIs minimised the loss of spatial detail and artefacts common in similar models.^14^ Yu *et al*^6^ also used a residual dense network to denoise LIE images in CCTA, greatly enhancing their suitability for visual analysis.

**Table 1:** Summary of recent major studies on image postprocessing

Author	Major work	Device	Technique
Matsuyama *et al*	Noise reduction	CCTA	DLIR (AiCE and PIQE)
Hong *et al*	Noise reduction	CCTA	DLIR (ClariCT.AI) combining with IR
Chang *et al*	Noise reduction	PC-CCTA	Pie-Net
Yu *et al*	Noise reduction	LIE images of CCTA	Residual dense network
Wang *et al*	Beam-hardening artefacts reduction	CCTA	DLIR (TrueFidelity) with HD scan mode
Kawai *et al*	Beam-hardening artefacts reduction	CCTA	DLIR (PIQE)
Maier *et al*	Motion artefacts reduction	CCTA	Deep PAMoCo
Caruso *et al*	‘Double-low’ scanning protocol	CCTA	DLIR (TrueFidelity)
Zhu *et al*	Image quality improvement in the overweight	CCTA	DLIR (TrueFidelity)
Zou *et al*	Influence of DLIR on plaque and stenosis analysis	CCTA	DLIR (PIQE)
Rossi *et al*	Influence of DLIR on CAC	ECG-gated non-enhanced cardiac CT	DLIR (TrueFidelity)
Xu *et al*	Influence of DLIR on CT-FFR	CT-FFR from CCTA	DLIR (AiCE)
Li *et al*	Coronary artery tree extraction	CCTA	U-Net
Javorszky *et al*	Plaque segmentation	CCTA	Attention U-Net
Wang *et al*	Coronary artery and vein segmentation	CCTA	AVDNet
Chen *et al*	Left atrial and ventricular blood pools segmentation	CCTA	U-Net
Lara-Hernandez *et al*	Phases registration	CT-MPI	Recursive cascade network

AiCE, Advanced Intelligent Clear-IQ Engine; AVDNet, Artery and Vein Disentanglement Network; CAC, coronary artery calcification; CCTA, coronary CT angiography; CT-FFR, CT-derived fractional flow reserve; CT-MPI, CT myocardial perfusion imaging.; DLIR, deep learning image reconstruction; HD, high-definition; IR, iterative reconstruction; LIE, late iodine enhancement; Deep PAMoco, deep partial angle-based motion compensation; PC-CCTA, photon-counting coronary CT angiography; Pie-Net, prior-information-enabled neural network; PIQE, Precise IQ Engine.

**Figure 2:**
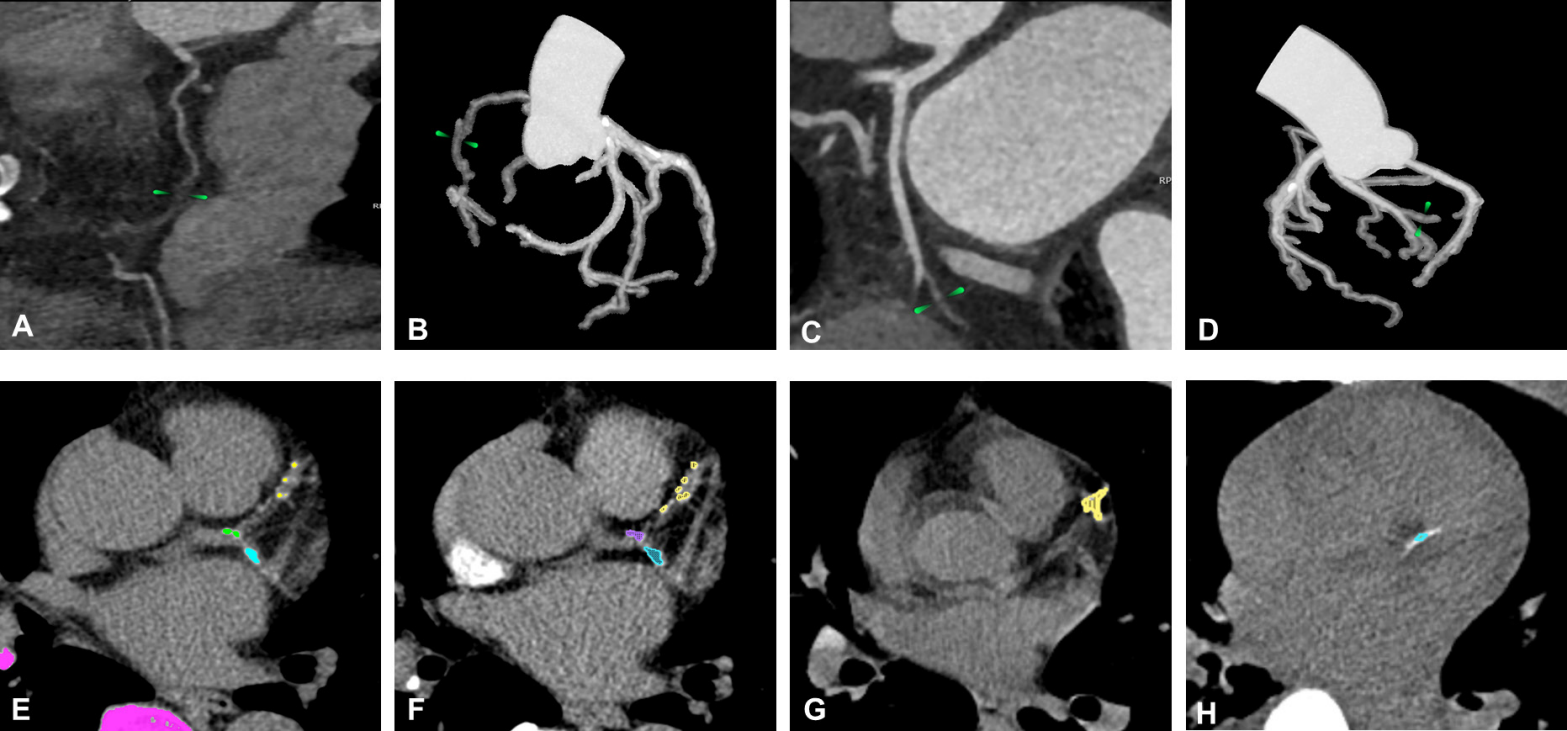
Common issues during coronary artery reconstruction. (**A, B**) Green arrows indicate motion artefacts from respiration or cardiac pulsation, causing blurred segments in the curved planar reformation (CPR) images and discontinuities in the segmented coronary artery tree. (**C, D**) Green arrows indicate veins with morphology and location similar to those of coronary arteries that may be misidentified as arteries, creating false segments in segmentation results. Schematic of manual and automated CAC measurement, where coloured regions represent calcifications from different coronary artery branches: (**E**) manual CAC annotation based on ECG-gated non-contrast cardiac CT; (**F**) DL-based CAC annotation from nongated images (eg, a single CT-MPI phase) yields results comparable to manual annotations from gated scans; (**G, H**) DL tools automatically identify CAC even in lower-quality images. CAC, coronary artery calcium; CT-MPI, CT myocardial perfusion imaging; DL, deep learning.

To further improve image quality by reducing artefacts, several studies have reported notable breakthroughs. Wang *et al*^9^ found that combining high-strength DLIR (DLIR-H) from General Electric (GE) with high-definition (HD) scan mode produced images with the smallest calcification diameters and significantly fewer beam-hardening artefacts while mitigating the typical noise increase associated with HD scans.^9^ Kawai *et al*^15^ demonstrated that, compared with IR, PIQE yielded superior overall image quality and clearer visualisation of the stent lumen and structure, thereby enhancing detection of in-stent restenosis, particularly in stents <3 mm in diameter.^15^ In addition to beam-hardening artefacts, motion artefacts from cardiac displacement during short-scan mode (figure 2) can also degrade image quality.^16^ Maier *et al*^16^ proposed deep partial angle-based motion compensation (Deep PAMoCo) to estimate and correct coronary artery motion. This DL model divides short-scan data into several consecutive angular segments, reconstructs each based on motion vector fields representing identical motion states, followed by aligning and combining these segments to obtain final images. Using partial-angle data allows Deep PAMoCo to capture subtle temporal variations in coronary motion that full-cycle reconstructions often blur. Moreover, DL-based motion vector estimation enables non-linear, data-driven modelling of cardiac motion, providing more accurate compensation relative to conventional registration-based methods. Thus, Deep PAMoCo effectively removes nearly all motion artefacts, regardless of coronary contrast, radius or motion amplitude. Postcorrection, Maier *et al* found that the mean CT value error along the coronary artery decreased from ~300 HU to 25 HU.^16^

Building on these improvements, researchers have explored broader AI applications. Leveraging DLIR’s strong denoising capability, Caruso *et al*^17^ implemented a ‘double-low’ scanning protocol, using low contrast agents and radiation doses, to apply GE’s DLIR-H in non-obese patients. Even with an 80-kVp and 1.4 iodine delivery rate (IDR) ‘double-low’ protocol, image quality exceeded that of the conventional 100-kVp and 1.8 IDR protocol.^17^ Similarly, Zhu *et al*^18^ found that in overweight patients, DLIR produced images with lower noise and higher contrast-to-noise ratio compared with standard IR, with DLIR-H performing best. Although increasing IR strength improved objective image quality, it also altered noise texture and lowered subjective image quality scores, whereas DLIR enhanced both objective and subjective image quality, especially in heavier patients.^18^ These findings indicate that DLIR could help reduce radiation exposure in all patients.

Some researchers have evaluated AI’s impact on CAD-related imaging parameters. Zou *et al*^19^ showed that using PIQE with a 60% dose reduction preserved agreement with conventional images for plaque volume, classification and stenosis analysis. Rossi *et al*^20^ compared coronary artery calcium (CAC)-related metrics across conventional reconstructions and varying GE DLIR strengths; they found that as DLIR strength increased, CACS and CAC volume decreased, whereas mass remained stable. This underestimation of CACS led to an 8% risk-category misclassification rate.^20^ Xu *et al*^21^ demonstrated that although AiCE improved CCTA image quality, CT-FFR values showed no significant differences compared with conventional reconstructions. These findings suggest that despite DLIR enhancing image quality, potential changes in CAD-related parameters, especially those linked to CAC, should be considered.

Beyond image reconstruction, automatic segmentation and registration of cardiac structures have become key research areas. Li *et al*^22^ applied U-Net to extract the coronary artery tree from CCTA. Javorszky *et al*^23^ developed a DL-based tool for coronary plaque segmentation on CCTA. The tool effectively segmented non-calcified plaques, which are difficult to differentiate from adjacent perivascular adipose tissue, achieving higher consistency with expert manual segmentations compared with mainstream approaches.^23^ However, similarities in intensity and proximity between coronary arteries and veins can still cause segmentation errors (figure 2), even in DL models.^24^ To address this, Wang *et al*^24^ introduced the Artery and Vein Disentanglement Network, which simultaneously incorporates arteries and veins in segmentation, yielding more robust and accurate vessel delineation in CCTA. Expanding beyond vessels, Chen *et al*^25^ used DL to automatically segment the left atrial and left ventricular blood pools in CCTA and reconstruct short-axis and long-axis views. In parallel, substantial progress has been made in automatic registration. Lara-Hernandez *et al*^4^ developed a DL model to register different CT-MPI phases, reducing left ventricular tissue displacement during registration and maintaining performance despite contrast variations, achieving processing times of only a few seconds.^4^

### AI and anatomical assessment

In anatomical analysis, extensive research has focused on the automatic assessment of plaques and stenosis. Han *et al*^26^ and Griffin *et al*^8^ showed that DL outperforms traditional methods in identifying varying degrees of stenosis and plaque types while markedly reducing diagnostic time. This approach helps limit the overestimation of stenosis severity caused by limited clinical experience. Brendel *et al*^27^ confirmed these findings in PC-CCTA, showing that with DL assistance, non-ultra high-resolution images achieved reliable stenosis assessments when expert-rated ultra high-resolution images were used as references. Nurmohamed *et al*^28^ further investigated AI-derived plaque and stenosis parameters for identifying ischaemia defined by FFR, finding that AI-based parameters outperformed both CT-FFR and nuclear MPI.^28^ Additionally, some studies examined factors influencing AI evaluation of plaques and stenosis. Xu *et al*^29^ found that plaque type did not affect diagnostic performance, whereas plaque length and CACS impacted accuracy. Tomasino *et al*^30^ demonstrated that AI-based quantitative plaque and stenosis analysis on CCTA yielded consistent results between systolic and diastolic phases, suggesting that although manual review of AI outputs remains necessary, the effect of cardiac phase is negligible. Beyond direct quantification, ML-assisted radiomics has been applied to plaque assessment. Radiomic analysis involves extracting numerous quantitative image features to characterise tissue phenotypes. Lin *et al*^31^ observed that different lesion types display distinct radiomic signatures and that integrating plaque and radiomic features helps identify optimal variable combinations that effectively detect culprit lesions causing myocardial infarction (MI). Incorporating these variables into ML models containing traditional features provided additional diagnostic value.^31^ Jin *et al*^32^ developed a DL model to segment coronary arteries and identify candidate plaques for downstream radiomic tasks, substantially reducing processing time and enabling efficient exploration of optimal radiomic features and ML classifiers.^32^

Parallel progress has been made in automating the quantification of other CAD-related structures. Several researchers have reported breakthroughs in AI-based CAC detection (figure 2). Eliminating the need for a separate calcium scoring CT scan by performing direct CAC measurements on CCTA can reduce radiation exposure.^33^ To achieve this, Mu *et al*^33^ created a DL-based calcium scoring tool for CCTA, which demonstrated strong correlation and consistent risk stratification compared with standard references. Their approach involved virtual non-contrast (VNC) images derived from spectral CT to annotate calcifications on CCTA. These VNC images, acquired concurrently with CCTA, closely resemble calcifications observed in ECG-gated non-contrast cardiac CT and clearly depict vascular areas with CT values below 130 HU, thereby significantly reducing the manual calcification annotation burden in CCTA.^33^ Zeleznik *et al*^3^ used DL to achieve strong agreement with manual CAC quantification in low-dose CT (LDCT) scans for lung cancer screening. Similarly, Zhou *et al*^34^ developed an AI model capable of accurately distinguishing proximal CAC from all detected calcifications in ECG-gated non-contrast cardiac CT, offering a novel method for CAC research.^34^ Cardiac-related adipose tissue has also been automatically quantified. Eisenberg *et al*^35^ and Miller *et al*^36^ applied DL to measure the volume and attenuation of epicardial adipose tissue (EAT) in ECG-gated non-contrast cardiac CT and LDCT, respectively. Additionally, Chan *et al*^37^ employed the AI-Risk algorithm to automatically derive the perivascular fat attenuation index (FAI) from CCTA and convert it into a risk score.

### AI and functional evaluation

**Figure 3:**
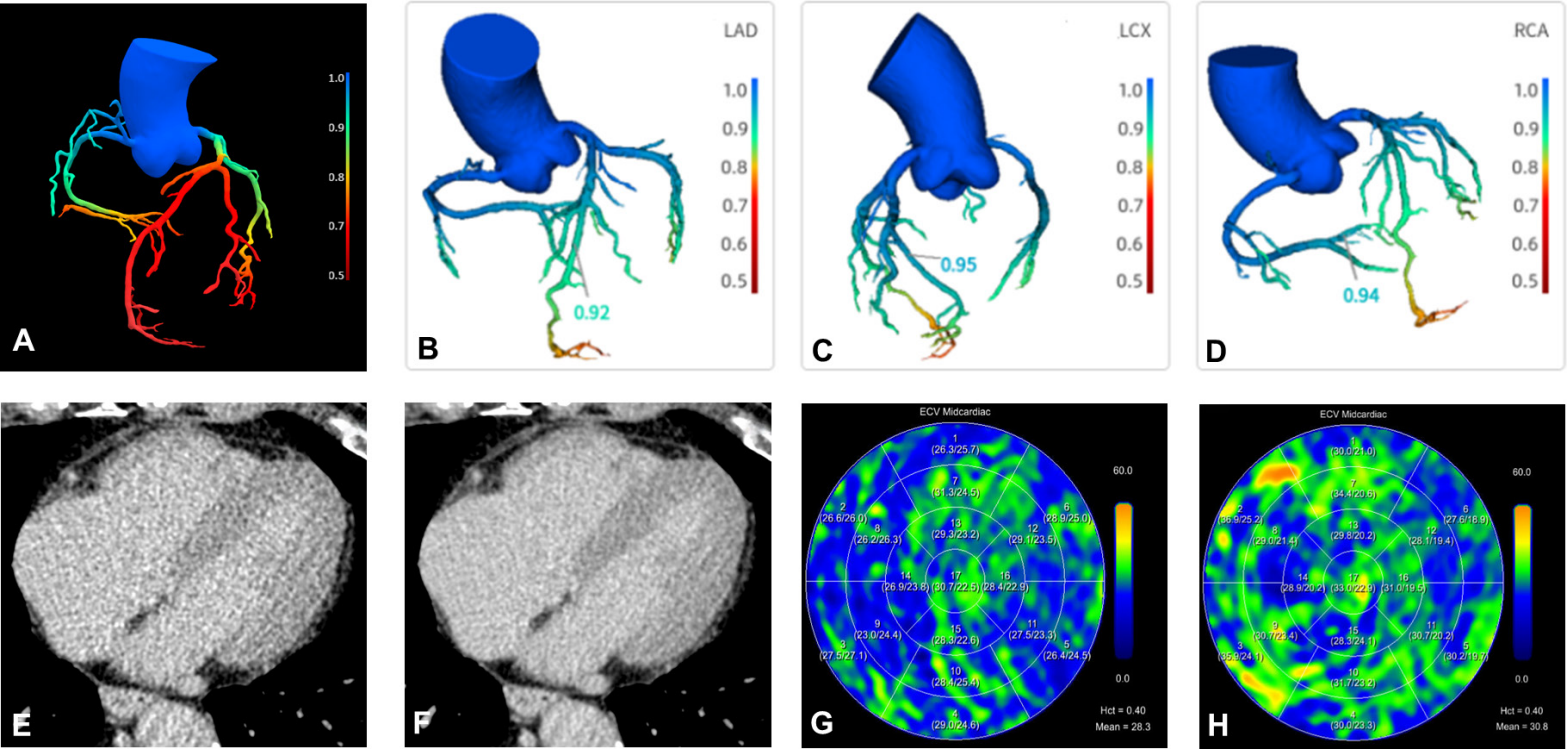
Schematic of DL-based CT-FFR and examples of LIE and ECV imaging. (**A**) Entire coronary artery tree. (B–D) Branch-specific quantification, with CT-FFR values labelled numerically at the indicated sites. Examples of LIE and ECV images: (**E, F**) CCTA LIE images before and after denoising; (**G, H**) ECV maps derived from LIE images showing a normal patient (mean<30) and an abnormal patient (mean>30). CCTA, coronary CT angiography; CT-FFR, CT-derived fractional flow reserve; DL, deep learning; ECV: extracellular volume; LIE, late iodine enhancement.

In automated functional assessment, most research has focused on CT-FFR. Compared with invasive coronary angiography (ICA), CCTA tends to overestimate stenosis severity, and anatomical narrowing does not always indicate haemodynamic significance, necessitating invasive FFR as the reference standard. To provide a non-invasive alternative, CT-FFR based on computational fluid dynamics was introduced and demonstrated good agreement with invasive FFR, although it remains computationally demanding.^5^ As shown in figure 3, DL-derived CT-FFR has now been developed, enabling assessments on conventional workstations.^7^ In a multicentre study, Coenen *et al*^7^ found that DL-based CT-FFR classified haemodynamically significant coronary stenosis more accurately compared with CCTA alone. Cardiac magnetic resonance (CMR) perfusion imaging can quantify myocardial blood flow (MBF), providing more direct visualisation of myocardial perfusion relative to FFR.^38^ Guo *et al*^38^ demonstrated that DL-based CT-FFR achieved diagnostic performance comparable to that of CMR perfusion in assessing haemodynamically significant stenosis.

Notably, CT-FFR techniques employed clinically continue to face limitations, including time-intensive manual processing.^39^ To overcome this, Guo *et al*^39^ developed a locally deployable CT-FFR system integrating automatic coronary lumen extraction and plaque segmentation. Compared with manual workflows, this approach reduced processing time threefold and decreased mouse clicks from ~60 to 1.^39^ CT-FFR also struggles within the ‘grey zone’, where clinical significance is uncertain. Improving CT-FFR accuracy in this range is essential for determining stent placement requirements.^40^ Although prior studies primarily used vascular imaging as input, Lee *et al*^40^ incorporated haemodynamic parameters, such as the oscillatory shear index, and patient biological data. Among eight AI models optimised and tested, the multilayer perceptron regression-based model showed the highest grey-zone accuracy.^40^ CT-FFR is further challenged by severe CAC and stent artefacts.^41^ To address this, Xu *et al*^41^ introduced subtraction CT-FFR, combining non-contrast and contrast-enhanced scans to digitally remove calcified or metallic artefacts, markedly improving diagnostic accuracy in evaluating calcified stenoses and stents compared with conventional CT-FFR.^41^

Several studies have examined additional factors influencing CT-FFR. Xu *et al*^21^ reported that although DLIR enhances image quality, it does not significantly alter CT-FFR values or diagnostic performance compared with IR. Tremamunno *et al*^42^ assessed the effect of VMI reconstruction energy levels on CT-FFR using PCCT, finding that 70 keV yielded the highest agreement with conventional CT, although no significant differences were observed across VMI levels and conventional CT.^42^ Koo *et al*^43^ and Tesche *et al*^44^ found that CACS has minimal influence on CT-FFR, whereas minimal lumen area and coronary artery stenosis length significantly affect its specificity and accuracy. Although diabetic patients often exhibit microcirculatory dysfunction and diffuse lesions that could affect CT-FFR, Xue *et al*^45^ demonstrated that diabetes does not reduce CT-FFR diagnostic accuracy.

**Figure 4:**
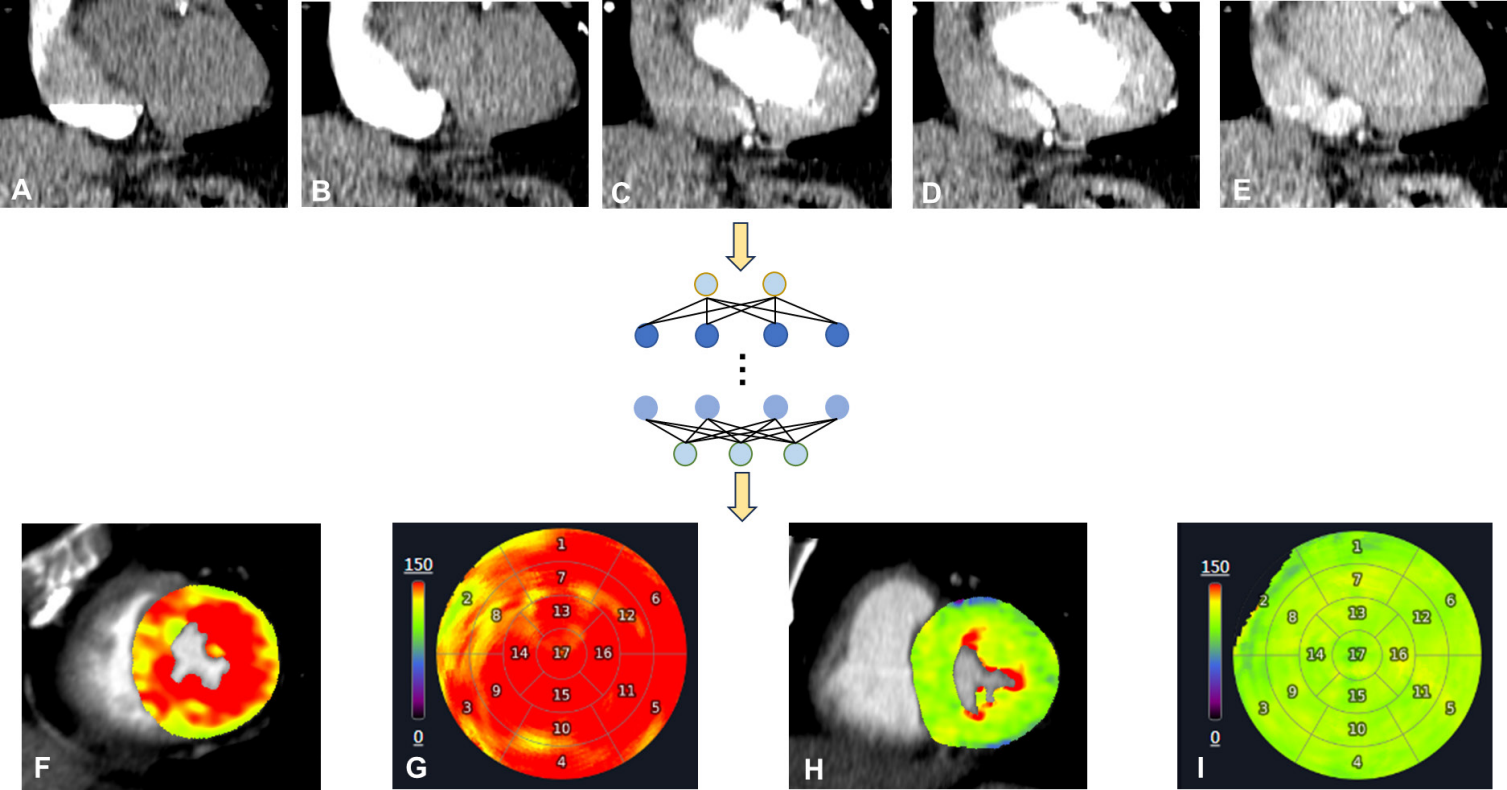
An overview of how CT-MPI data is processed by DL. (A–E) CT-MPI acquires~10–15 phases during image acquisition; cardiac motion causes position variation across phases. Image registration is required before analysis. DL tools can directly map raw data to final parametric maps. (**F, G**) MBF short-axis pseudocolor map and 17-segment bull’s-eye plot of a normal patient; (**H, I**) MBF short-axis pseudocolor map and 17-segment bull’s-eye plot of a patient with ischaemia (dark green area). CT-MPI, CT myocardial perfusion imaging; DL, deep learning; MBF, myocardial blood flow.

Beyond CT-FFR, AI applications in other functional parameters remain limited. MBF derived from CT-MPI can reliably detect myocardial ischaemia, but the absence of AI-assisted tools restricts its clinical use.^46^ Yu *et al*^46^ developed an AI-based system for automated MBF quantification, achieving high consistency with manual results (figure 4). This AI tool also automatically determines the ischaemic myocardial volume percentage, enhancing ischaemia visualisation.^46^ Lee *et al*^47^ combined CT-FFR with plaque biomechanical factors, specifically wall shear stress (the tangential frictional force exerted by blood flow on the vessel wall) and axial plaque stress (the longitudinal mechanical stress within the plaque induced by pulsatile blood pressure), to identify plaques with acute coronary syndrome risk. However, DL has not been used to compute these biomechanical parameters.^47^ Yu *et al*^6^ applied DL to denoise LIE images, producing more accurate extracellular volume measurements and improving myocardial fibrosis detection (figure 3).

### AI and treatment guidance

In treatment decision support, CT-FFR has become a valuable tool for determining invasive intervention requirements. Qiao *et al*^48^ reported that in patients with stable angina, integrating CT-FFR into ICA modified the initial treatment strategy in 14.9% of cases. Moreover, reserving ICA for patients with unfavourable CT-FFR results reduced ICA rates by 54.5% and percutaneous coronary intervention (PCI) rates by 4.4%.^48^ Similarly, Yang *et al*^49^ found that, compared with standard care, patients with stable angina in the CT-FFR group had fewer ICAs performed, even in the absence of obstructive CAD, and fewer missed ICAs when obstructive CAD was present. The CT-FFR group also showed a higher PCI rate, suggesting that CT-FFR enables more accurate patient selection and reduces unnecessary medical expenses.^49^

When choosing between PCI and coronary artery bypass grafting, anatomical complexity remains a critical criterion. The synergy between PCI with taxus and cardiac surgery (SYNTAX) score is considered the standard for assessing anatomical complexity in multivessel CAD and guiding treatment decisions.^50^ When combined with CT-FFR, the SYNTAX score becomes the functional SYNTAX score (FSS), which effectively reclassifies patients into lower-risk categories and reduces unnecessary vascular interventions.^51^ However, compared with CT-FFR, CT-MPI demonstrates superior diagnostic accuracy in detecting haemodynamically significant CAD. Assessing this, Dai *et al*^50^ confirmed that the FSS derived from CT-MPI strongly correlates with the traditional ICA-based FSS. Notably, CT-MPI FSS reclassified 22.7% of patients from the intermediate-to-high risk group to the low-risk group, refining risk stratification. Additionally, in patients whose CT-FFR results were limited by severe calcification, the CT-MPI-based FSS accurately identified intermediate-to-high risk patients, underscoring its value in treatment plans.^50^

**Figure 5:**
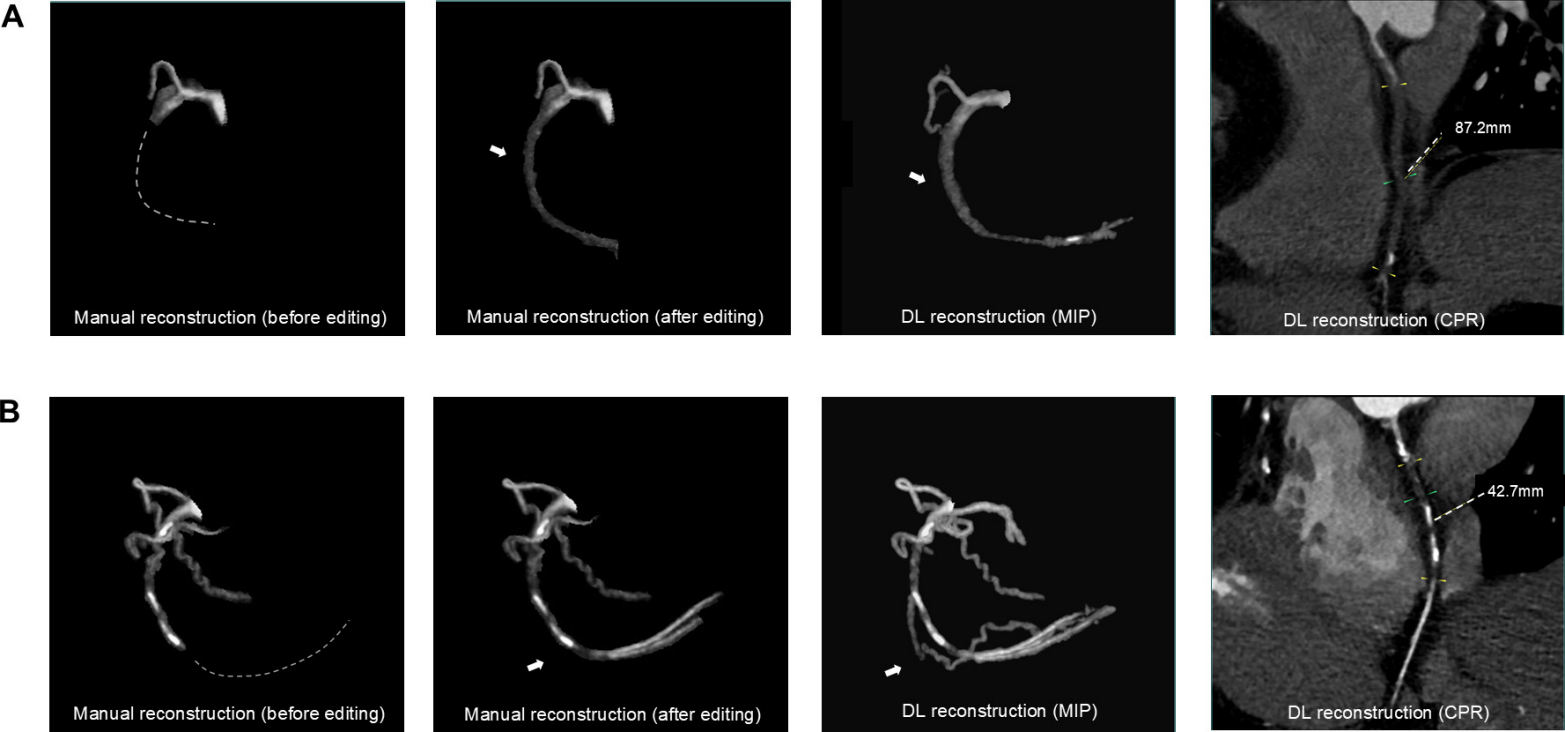
Comparison of manual and DL–based coronary CTO reconstruction. (**A**) Case 1: (from left to right) manual reconstruction before editing shows the failure of the commercial workstation to extract the occluded segment (dashed lines); manual reconstruction after editing with the occluded segment manually added using a multipoint vessel-tracking tool (arrow); DL-based reconstruction (MIP) showing automatic extraction of the entire CTO pathway (arrow) with more small branches; and DL-based CPR image showing the reconstructed CTO. (**B**) Case 2: same sequence of panels as in (**A**), illustrating another representative case. CPR, curved-planar reformation; CTO, chronic total occlusion; DL, deep learning; MIP, maximum intensity projection.

Chronic total occlusion (CTO) lesions, that is, complete coronary artery blockages persisting for at least 3 months, are characterised by complex morphology and limited collateral flow. They are frequently observed in patients with suspected obstructive CAD and often require ICA; however, PCI for CTOs remains technically challenging. Compared with ICA, CCTA provides superior visualisation of CTO features, potentially improving revascularisation success. Nonetheless, reconstructing CTOs using conventional CCTA is time-consuming because occluded segments lack contrast enhancement and are not automatically detected by current commercial postprocessing tools.^52^ To overcome this limitation, Li *et al*^52^ developed a DL-based CTO reconstruction method. As shown in figure 5, their approach achieved automated segmentation and reconstruction in 95% of CTO lesions without manual correction, representing a marked improvement over the 48% success rate of conventional techniques, while greatly reducing postprocessing time and maintaining strong agreement with manual evaluations.^52^ Building on this, Zhou *et al*^53^ demonstrated that DL not only enables accurate CTO reconstruction and analysis but also predicts the likelihood of successful guidewire crossing within 30 min and overall PCI success, significantly outperforming traditional manual scoring systems. These findings highlight DL’s efficiency and potential to enhance clinical decision-making in CTO interventions.

### AI and risk stratification

AI has played a major role in advancing risk stratification (table 2). Much of this work emphasises prognostic evaluation based on DL-derived parameters, particularly vascular and plaque-related metrics. One of the most practical applications is the automated quantification of CACS. In a large-scale cohort study, Zeleznik *et al*^3^ demonstrated that automated CACS obtained from ECG-gated non-contrast cardiac CT or LDCT independently predicts cardiovascular events. Zhou *et al*^34^ achieved a notable breakthrough with a 14-year follow-up study on major adverse cardiovascular events (MACE), demonstrating that incorporation of proximal CAC into conventional scoring systems improves risk prediction. Expanding beyond calcium-based measures, some studies have integrated additional plaque parameters. In an international multicentre study with a median 4.7-year follow-up, Lin *et al*^2^ showed that a DL-based automated plaque assessment tool predicted MI: after adjusting for obstructive stenosis and clinical risk scores, a total plaque volume ≥238.5 mm³ remained a significant MI predictor.^2^ In a study with a median follow-up of 10.3 years, Nurmohamed *et al*^28^ reported that an AI-based quantitative CCTA tool, integrating stenosis, plaque burden and vascular morphology, retained predictive power for MACE after adjustment for clinical risk factors and obstructive stenosis. In another study, Qiao *et al*^48^ demonstrated over a 26-month median follow-up that a CT-FFR value ≤0.80 was significantly associated with MACE, outperforming severe stenosis identified through ICA and CCTA. Building on this, Chen *et al*^54^ conducted a study with a median follow-up of 5.2 years, revealing that improvements in distal CT-FFR correlated with decreased high-risk plaque features, increased lumen volume and higher remodelling index values, whereas deterioration in distal CT-FFR was linked to greater luminal stenosis, more medium-density calcified plaques and elevated total cholesterol levels. These findings clarify the mechanisms underlying CT-FFR’s prognostic value for MACE. Synthesising prior results, Dundas *et al*^55^ combined anatomical and functional vascular parameters, showing in a 1-year follow-up that AI-enabled quantitative CCTA plaque volume added prognostic value for MACE beyond luminal stenosis and CT-FFR. Notably, total plaque volume>563 mm³ was associated with greater MACE risk.

**Table 2:** Summary of AI-assisted risk stratification

Author	Main conclusion
Zeleznik *et al*	DL-based CACS from ECG-gated non-contrast cardiac CT or LDCT is an independent predictor of cardiovascular events
Zhou *et al*	Incorporating AI-based proximal CAC into conventional scoring enhances prediction of MACE
Lin *et al*	A total DL-based plaque volume of≥238.5 mm³ is a significant predictor of MI
Nurmohamed *et al*	Stenosis, plaque, vascular morphology assessed by AI-QCT tool are predictors of MACE
Qiao *et al*	DL-based CT-FFR≤0.80 is significantly associated with MACE, surpassing severe stenosis
Chen *et al*	The change in distal DL-based CT-FFR is associated with stenosis and plaque features
Dundas *et al*	DL-based plaque volume provides incremental prognostic value for MACE beyond stenosis and CT-FFR, with a total volume＞563 mm³ associated with a higher risk
Miller *et al*	Both the DL-based volume and attenuation of EAT are independently associated with death or MI
Brandt *et al*	DL-based EAT volume has incremental value in predicting ischaemia, comparable to CT-FFR
Eisenberg *et al*	A DL-based EAT volume of≥113 cm³ is the strongest predictor of MACE, having correlations with specific serum biomarkers
Hoshino *et al*	A DL-based FAI cut-off value of −73.1 HU optimally predicts MACE, having association with vessel and plaque features
Chan *et al*	AI-based FAI and risk scores enhance risk assessment in patients with non-obstructive CAD
Oikonomou *et al*	PCAT radiomic features enhance the prediction of MACE, having correlation with specific gene expressions
Huang *et al*	DL-based PCAT features outperform existing methods in predicting acute coronary syndrome
Tesche *et al*	ML-based model integrating clinical and plaque features improves the prediction of MACE
Pezel *et al*	ML-based model integrating CMR perfusion and CCTA data enhances the prediction of MACE
Lin *et al*	ML-based model is able to predict ischaemia defined by MBF using purely anatomical features

AI, artificial intelligence; AI-QCT, AI-based quantitative CCTA; CAC, coronary artery calcification; CACS, coronary artery calcium scores; CAD, coronary artery disease; CCTA, coronary CT angiography; CMR, cardiac magnetic resonance; CT-FFR, CT-derived fractional flow reserve; DL, deep learning; EAT, epicardial adipose tissue; FAI, fat attenuation index; HU, Hounsfield Units; LDCT, low-dose CT; MACE, major adverse cardiovascular events; MBF, myocardial blood flow; MI, myocardial infarction; ML, machine learning; PCAT, pericoronary adipose tissue.

Another major focus of DL-based automated quantification is adipose tissue evaluation. In a study with a median follow-up of 2.7 years, Miller *et al*^36^ found that EAT volume and attenuation independently predicted increased risk of death or MI. Brandt *et al*^56^ showed that adding EAT volume to plaque analysis improved prediction of lesion-specific ischaemia, achieving accuracy comparable to that of CT-FFR and suggesting EAT’s utility in identifying haemodynamically significant coronary lesions. Eisenberg *et al*^35^ extended this work in a 14-year follow-up integrating serum biomarker data, revealing that increased EAT volume and reduced attenuation independently predicted higher MACE risk even after adjusting for traditional risk factors. An EAT volume ≥113 cm³ exhibited the strongest predictive power. Moreover, EAT characteristics correlated with specific serum biomarkers, including C reactive protein, myeloperoxidase and adiponectin. Pericoronary adipose tissue (PCAT) and its derived perivascular FAI have also garnered attention. In a study with a median follow-up of 792 days, Hoshino *et al*^57^ identified −73.1 HU as the optimal DL-based FAI threshold for predicting MACE. Higher FAI values correlated with larger vessel diameters, increased plaque volumes and high-risk plaque features. Chan *et al*^37^ further validated the FAI’s prognostic value in non-obstructive CAD, showing that AI-derived FAI and risk scores enhanced risk assessment in this population, where management remains unclear, thereby improving prognosis. Beyond basic metrics, such as fat volume or attenuation, Oikonomou *et al*^1^ used a radiomic approach to characterise PCAT. Over a 5-year follow-up, they found that PCAT’s radiomic features significantly correlated with the expression of genes linked to inflammation, fibrosis and vascular remodelling, notably enhancing MACE prediction compared with conventional methods.^1^ Addressing radiomics limitations, Huang *et al*^58^ developed a DL framework that more accurately captured PCAT characteristics, outperforming existing advanced models in predicting acute coronary syndrome.

In addition to automated imaging feature extraction, ML classifiers have also replaced traditional predictive models. Over a median 5.4-year follow-up, Tesche *et al*^59^ demonstrated that ML models integrating clinical and CCTA-derived plaque features significantly improved MACE prediction and risk stratification. Capitalising on ML’s advantages, Pezel *et al*^60^ performed a study with a median follow-up of 7 years, integrating CMR perfusion parameters into CCTA data and showing that this multimodal model further enhanced MACE prediction. Lin *et al*^61^ introduced an ML model to predict ischaemic lesions defined by positron emission tomography–derived MBF, allowing functional impairment prediction solely from anatomical data. This model achieved an area under the curve surpassing visual stenosis analysis and comparable to CT-FFR. Among analysed variables, per cent diameter stenosis and low-density non-calcified plaque volume contributed most to the model’s predictive accuracy.^61^

#### Summary

AI has been successfully applied to enhance image quality across various cardiac CT devices and sequences, enabling radiation dose reduction and advancing automatic segmentation and registration of cardiac structures. These developments have significantly enhanced efficiency within radiology departments. Furthermore, AI has enabled automated assessment of CAD-related anatomical parameters and CT-FFR, streamlining diagnostic workflows. In treatment planning, AI provides detailed anatomical and functional insights that support preoperative decision-making for interventional procedures. In prognosis, AI-driven quantification of imaging parameters and integration of clinical factors have strengthened the predictive power of cardiac CT in assessing CAD risk.

Despite these advances, several important challenges remain. Radiologists and other clinicians applying AI-based image reconstruction must carefully select appropriate AI reconstruction models and remain aware that image style may alter anatomical details. When applying automatic segmentation and anatomical analysis tools, outputs should be fine-tuned based on the specific clinical context rather than relying solely on AI outputs. For treatment planning and prognostic applications, selecting suitable tools for decision support while considering AI model interpretability is crucial. For researchers, further exploration of AI’s potential across broader clinical scenarios is necessary, particularly focusing on developing novel functional indicators, supporting decision-making for diverse diseases and building more comprehensive risk prediction models. The technical and financial barriers to AI tool development and deployment should also be addressed to ensure accessibility and equitable use. Beyond these technical and clinical considerations, the broader adoption of AI in cardiac imaging raises ethical and regulatory concerns, including data privacy, patient consent and algorithmic bias. Addressing these issues is essential to achieve safe, transparent and equitable clinical implementation of AI.

Overall, this review of recent advances in AI applications in CAD-related CT will help guide clinical practitioners in integrating these cutting-edge technologies into practice. Additionally, it offers researchers perspectives on potential directions for future investigations. Ultimately, the goal is to further advance CAD diagnosis and treatment through the effective implementation and use of AI.

## Data Availability

Data sharing not applicable as no datasets generated and/or analysed for this study.
